# Genetics of Ascites Resistance and Tolerance in Chicken: A Random Regression Approach

**DOI:** 10.1534/g3.112.002311

**Published:** 2012-05-01

**Authors:** Antti Kause, Sacha van Dalen, Henk Bovenhuis

**Affiliations:** Wageningen University and Research Centre, Animal Breeding and Genomics Centre, De Elst 1, 6708 WD, Wageningen, The Netherlands

**Keywords:** disease, heritability, genotype-by-environment interaction, genetic trade-off, reaction norm, resilience

## Abstract

Resistance and tolerance are two complementary mechanisms to reduce the detrimental effects of parasites, pathogens, and production diseases on host performance. Using body weight and ascites data on domesticated chicken *Gallus gallus domesticus*, we demonstrate the use of random regression animal model and covariance functions to estimate genetic parameters for ascites resistance and tolerance and illustrate the way individual variation in resistance and tolerance induce both genotype re-ranking and changes in variation of host performance along increasing ascites severity. Tolerance to ascites displayed significant genetic variance, with the estimated breeding values of tolerance slope ranging from strongly negative (very sensitive genotype) to weakly negative (less sensitive). Resistance to ascites had heritability of 0.34. Both traits are hence expected to respond to selection. The two complementary defense strategies, tolerance and resistance, were genetically independent. Ascites induced changes to the correlations between ascites resistance and body weight, with the genetic correlations being weak when birds were ascites-free but moderately negative when both healthy and affected birds were present. This likely results because ascites reduces growth, and thus high ascites incidence is genetically related to low adult body weight. Although ascites induced elevated phenotypic and genetic variances in body weight of affected birds, heritability displayed negligible changes across healthy and affected birds. Ascites induced moderate genotype re-ranking in body weight, with the genetic correlation of healthy birds with mildly affected birds being unity but with severely affected birds 0.45. This study demonstrates a novel approach for exploring genetics of defense traits and their impact on genotype-by-environment interactions.

Resistance and tolerance are two complementary defense mechanisms against pathogens and parasites. Resistance is the host trait that prevents infection in the first place or reduces the performance of a pathogen on a host; both factors reduce the pathogen burden within a host individual. Tolerance to infections, in turn, is defined as the ability of the host to limit the impact of a given pathogen burden on host health, performance, and ultimately on fitness (Clunies-Ross 1932; Painter 1958; Simms and Triplett 1994; Simms 2000). In farm animal science, tolerance is sometimes called resilience (Riffkin and Dobson 1979; Albers *et al.* 1987; Bisset and Morris 1996). In addition to pathogens, tolerance can be assessed against abiotic factors such as heavy metals, temperature, frost damage, or against production diseases causing damage to body tissues (Ravagnolo and Misztal 2000a,b; Schat *et al.* 2002; Agrawal *et al.* 2004; Kause 2011; Bloemhof *et al.* 2012). In farmed plant and animal species, both increased resistance and tolerance serve as ways to insure global food security.

Tolerance can be analyzed as a reaction norm in which host performance (on *y*-axis) is regressed against an increasing pathogen burden or abiotic factor (on *x*-axis) (Simms 2000). Genetic variance in regression slopes is hence the genetic variance for tolerance. When there is genetic variation for tolerance, heritability for host performance (*e.g.*, growth, reproduction, or fitness) can potentially change across increasing pathogen burden (Kause 2011). For instance, diverging reaction norms imply an existence of genotype-by-environment interaction that creates increasing genetic variance for host performance. Diseases are indeed known to induce changes in heritability of host performance traits (Charmantier *et al.* 2004; Vehviläinen *et al.* 2008; Lewis *et al.* 2009). Moreover, crossing tolerance reaction norms imply genotype-by-environment interaction that creates genotype re-ranking in host performance. Genotype-by-environment interactions together with environment-dependent selection forces promote divergent genetic responses in different environments (Falconer 1952; Joshi and Thompson 1995; Kause *et al.* 2001). Despite the large number of studies dealing with the changes induced by biotic (*e.g.*, diet) and abiotic factors in general (Hoffmann and Merilä 1999; Kause and Morin 2001; Charmantier and Garant 2005), there has been only a limited focus on infection-induced changes in genetic parameters and genetic responses to selection.

Kause (2011) introduced the use of random regressions and covariance functions for genetic analysis of tolerance. These methods allow the estimation of genetic variance for resistance and tolerance and their genetic correlations with other traits in a single multitrait analysis. Covariance functions allow the estimation of genetic variance for host performance at any point along the increasing pathogen burden trajectory as well as the degree of genotype re-ranking between any of the points, providing novel means to analyze infection-induced genotype-by-environment interactions in host performance (Kirkpatrick *et al.* 1990; Calus *et al.* 2004; Kause 2011). In the present study, these methods were applied to the genetic analysis of ascites resistance and tolerance in domesticated chicken *Gallus gallus domesticus*.

Ascites is a metabolic disorder in individuals that fail to fully supply the demand for oxygen in their bodies because of a mismatch between cardiopulmonary system output and the demands of the body (Decuypere *et al.* 2000). Ascites resistance is the ability of a bird to prevent ending up in a physiological state in which there is a discrepancy between oxygen uptake and oxygen requirement, resulting in overloading of the cardiopulmonary system. Chicken compensate for hypoxia by circulating more blood through heart, resulting in an enlarged right ventricular. Consequently, heart ratio, the ratio of right ventricular weight to total heart weight, is used as an indicator of ascites resistance, that is, whether chicken have ascites or not (Wideman *et al.* 1998; Balog *et al.* 2003; Zerehdaran *et al.* 2006; and references therein). Furthermore, the occurrence of ascites is associated with reduced growth, hepatic damage, transduction of fluid into abdominal body cavity, and occasionally death (Julian 1998). Ascites tolerance is the ability of a bird to limit the consequences of a discrepancy between oxygen uptake and oxygen requirement on reproduction, growth, and fitness. Under commercial production conditions, the incidence of ascites is low but significant enough to cause reduced animal welfare (Julian 1998; Decuypere *et al.* 2000). To effectively study ascites, its incidence can be elevated by exposing birds to low temperature and increased CO_2_ levels.

In this study, we defined ascites tolerance in chicken as a reaction norm of body weight along an increasing ascites severity, measured as the heart ratio. This approach follows the definition by Simms (2000) with the difference that we are dealing with a production disease, not pathogens or parasites. The concepts of resistance and tolerance apply equally well to ascites, but the standard host-pathogen co-evolutionary interactions (Mauricio *et al.* 1997; Rausher 2001; Bishop and MacKenzie 2003; Best *et al.* 2008) cannot be applied to production diseases because ascites does not evolve in response to host evolution. Here we demonstrate the merit of the suggested novel statistical methods (Kause 2011) for genetic analysis of tolerance. We first estimated genetic variances and genetic correlations for tolerance, resistance, and growth performance. These estimates reflect whether a lack of genetic variance or genetic trade-offs limit genetic improvement of these traits. Second, we examined whether ascites induces genotype-by-environment interactions across healthy and affected birds, in terms of variance changes and genotype re-ranking in body weight.

## Methods

The experiment was conducted at the facilities of Hendrix Genetics/Cobb Europe BV, located in Boxmeer, The Netherlands. The experiment was performed by licensed and authorized personnel under approval of Cobb Europe BV.

### Population structure

The experimental offspring population consisted of 7722 purebred White Plymouth Rock broilers, of which 3745 were males and 3977 females. They descended from 83 sires and 788 dams. Each sire was mated to an average of 15.7 dams (range, 5-28 dams), and each dam was mated to an average of 1.65 sires (range, 1-3 sires). Sire−family sizes ranged between 22 and 209, with an average of 93 offspring per sire. There were 2677 ancestors in 25 generations, and they did not have any trait records. Eight sires with fewer than 20 offspring were removed from the data because large family sizes are needed to avoid biased genetic correlation between tolerance slope and intercept and biased genetic variance estimate in tolerance slope (Mauricio *et al.* 1997; Tiffin and Rausher 1999; Kause 2011).

### Rearing procedure

Eggs laid by all the dams were collected until a batch with a total of approximately 1600 chicks were obtained, and a total of five batches were produced. The eggs were individually numbered along with their dam code and transferred to a common brooding machine. At the day of hatching, the chicks were sexed, individually wing tagged, and group housed in two stables with 14 birds/m^2^. During the whole experiment, birds had free *ad libitum* access to water and commercial feed with 12.970 KJ/kg. Birds were exposed to 23 hr of light per day.

To challenge birds to ascites, they were kept under cold conditions and increased CO_2_ levels. At the time of hatching, temperature was held at 30°. During the successive 11 days temperature was gradually reduced to 12°. Thereafter, the temperature was kept at 12° until week 7 when the experiment was terminated. To increase CO_2_ level in the stables to approximately 1500 ppm, the ventilation was reduced from day 11 onwards. Except for the CO_2_ level and temperature, rearing conditions closely resembled the commercial practice. Survival from the first body weight recording at week 2 to the end of the experiment was 91.0%.

The experiment with the two stables was repeated in five successive batches of offspring. Each sire and dam had offspring in an average of 3.54 (range, 1-5) and 2.58 (range, 1-5) batches, respectively.

### Trait definitions

Birds were individually weighed at 2 (trait: BW2) and 7 weeks of age (BW7). After BW7 recording, birds were euthanized using CO_2_. A postmortem examination was performed to measure heart ratio: the percentage of right ventricle weight from total heart weight (RATIO). RATIO was recorded by 11 different people trained for cutting. Using random regressions, we defined two additional traits: 7-week body weight (BW7) of ascites-free birds (trait: INTERCEPT) and tolerance slope for BW7 (SLOPE).

Heart ratio is generally agreed to be an indicator trait for ascites, and birds with heart ratio greater than 27% to 30% are ascitic (Wideman *et al.* 1998; Balog *et al.* 2003; Zerehdaran *et al.* 2006; and references therein). It should be noted, however, that there may be some birds that have ascites but their heart ratio resembles that of a more healthy bird (and vice versa). This could be, for instance, because their heart is tolerant against ascites-induced hypoxia. Thus, it is possible that heart ratio, a measure of resistance, may in fact involve a component of tolerance in it. It is expected that body weight is reduced only in affected animals with heart ratio greater than 27% to 30%, whereas in healthy animals no relationship between heart ratio and body weight should exist. To make such a plateau-linear model (Ravagnolo and Misztal 2000a,b), heart ratio equal to or below 29% was coded as zero, and heart ratio greater than 29% was coded as: RATIO-29%. This corrected heart ratio (trait: RATIOPlat) was then used as an *x*-axis in the tolerance analysis. The plateau-linear tolerance regression model with RATIOPlat (Akaike Information Criteria [AIC] = 3654; Bayesian Information Criteria [BIC] =3674) fitted the data better than the linear model in which RATIO was used as the *x*-axis (AIC = 3842; BIC =3866). The genetic model 3 provided in the next section was used in this comparison for both models. Moreover, AIC and BIC were used to find the best-fitting threshold of 29% within the potential heart ratios of 27% to 30%. It should be noted that RATIO is used here as the continuous-scale measure of ascites resistance, whereas RATIOPlat is used only as the *x*-axis in the tolerance analysis (yet their genetic analysis produced similar results). Sample size was 7710, 7039, and 6991 birds for BW2, BW7, and RATIO, respectively. The trait means are given in Table 1 and File S1.

**Table 1:** Trait means (units in brackets), genetic variances (*V*_G_), maternal variances (*V*_M_), heritabilities (*h*^2^), maternal effect ratios (*m*^2^), and their standard errors (± SE)

	BW2 (g)	BW7 (g)	RATIO (%)	INTERCEPT (g)	SLOPE*^a^* (g/%)
Mean	248	2075	28.2%	2080	-14.6
*V*_G_ ± SE	309 ± 57.01	12952 ± 2865	15.1 ± 2.559	10640 ± 2540	57.8 ± 37.47
*V*_M_ ± SE	59.3 ± 12.66	2913 ± 778	1.17 ± 0.518	2771 ± 769	23.0 ± 23.11
*h*^2^ ± SE	0.33 ± 0.05	0.18 ± 0.04	0.34 ± 0.05	−	−
*m*^2^ ± SE	0.06 ± 0.01	0.04 ± 0.01	0.03 ± 0.01	−	−
*V*_P_	946	73479	46.72	−	−

a *V*_G_ and *V*_M_ from the model including the fixed BW2 covariate were 59.34 ± 33.0 and 6.50 ± 18.9, respectively.

### Genetic analysis

All analyses were performed with multitrait animal models using ASReml (Gilmour *et al.* 2006). The animal model takes into account all the relationships between individuals in the pedigree. Body weights BW2 and BW7 were analyzed with the animal “trait mean” model:

(1) $$y_{ij}\;=\;\mu_{i}+\mathrm{ani}m_{j}+\mathrm{da}m_{k}+\mathrm{GENDE}R_{l}+\mathrm{BATC}H_{m}\;\;\;\times \mathrm{STABL}E_{n}+\mathrm{AG}E_{p}+\mathrm{erro}r_{ijklmnp,}$$

and RATIO with the animal “trait mean” model:

(2) $$y_{ij}=\mu_{i}+\mathrm{ani}m_{j}+\mathrm{da}m_{k}+\mathrm{GENDE}R_{l}+\mathrm{BATC}H_{m}\;\times \mathrm{STABL}E_{n}+\mathrm{AG}E_{p}+\mathrm{PERSO}N_{q}+\mathrm{erro}r_{ijklmnpq,}$$

where *y_ij_* is an observation of a trait *i* (*i* = 1-3) for the *j*th individual (*j* = 1-number of individuals); µ*_i_* is the mean for trait *i*; anim*_j_* is the random animal genetic effect with a pedigree; dam*_k_* is the random dam effect without a pedigree (*k* = 1-788); GENDER*_l_* is the fixed effect of gender (*l* = 1-2); BATCH*_m_*×STABLE*_n_* is the fixed interaction of rearing batch (*m* = 1-5) and stable (*n* = 1-2); AGE*_p_* is the fixed effect of animal age when a trait *i* was recorded (for BW2 *p* = 12-13 days, for BW7 *p* = 45-46, for RATIO *p* = 46-48); and PERSON*_q_* is the fixed effect of a cutter of a heart (*q* = 1-11), and error is the random error.

Ascites tolerance was analyzed with the random regression animal model:

(3) $$\begin{array}{l} y_{j}=b_{0j}+b_{1j}+b_{0k}+b_{1k}+\mathrm{GENDE}R_{l}\;+\mathrm{BATC}H_{m}\times \mathrm{STABL}E_{n}+\mathrm{AG}E_{p}+\mathrm{PERSO}N_{q}+b_{0}+b_{1}+\;\\ b_{1}\mathrm{BATC}H_{m}\times \mathrm{STABL}E_{n}+b_{1}{\mathrm{PERSON}}_{q}+\mathrm{erro}r_{ijklmnpq,} \end{array}$$

where *y_j_* is BW7 of an animal *j*; b_0_*_j_* is the random intercept of an animal *j*; *b*_1_*_j_* is the random tolerance slope of BW7 on RATIOPlat for an animal *j*; b_0_*_k_* is the random intercept of dam *k*; *b*_1_*_k_* is the random tolerance slope of BW7 on RATIOPlat for dam *k*; *b*_0_ is the fixed population mean intercept; *b*_1_ is the fixed population mean tolerance slope; *b*_1_BATCH*_m_*×STABLE*_n_* is the fixed tolerance slope for each *m* batch and *n* stable; and *b*_1_PERSON*_q_* is the fixed tolerance slope for each cutter. To avoid heterogeneous error variance inflating genetic variance in slope (Lillehammer *et al.* 2009), residual variance was estimated within five RATIOPlat classes along the *x*-axis. The classes were defined as: RATIOPlat = 0, 0–5, 5–10, 10–15, and >15.

Animal and sire solutions from these mixed models are estimated breeding values (EBVs) quantifying the genetic level of individuals for a trait.

An additional model was run in which BW2 was included into the model 3 as a fixed covariate (*i.e.*, a regression term). This accounts for a possibility that ascites might have been more common among initially fast or slow growing individuals. In fact, BW2 had very weak genetic and phenotypic correlations with RATIO (*r*_G_ = 0.12 ± 0.12, *r*_P_ = 0.04 ± 0.02; Table 2), and hence in our data ascites incidence was independent of initial growth. When initial host performance and resistance are correlated, for example, pathogens are nonrandomly distributed across individuals, and this is not accounted for in the statistical model, biased tolerance genetic variance is estimated (Kause 2011).

**Table 2:** Genetic (upper panel, above diagonal), maternal (upper panel, below diagonal), and phenotypic correlations (lower panel) and their standard errors (± SE)

	BW2	BW7	RATIO
Genetic and maternal correlations			
BW2		0.60 ± 0.09	0.12 ± 0.12
BW7	0.78 ± 0.10		−0.33 ± 0.12
RATIO	−0.14 ± 0.22	−0.35 ± 0.22	
Phenotypic correlations			
BW2		0.47 ± 0.01	0.04 ± 0.02
BW7			−0.23 ± 0.02

Variances and variance ratios were considered significant when 0.98 times their standard error did not include zero (one-tailed test). Correlations were considered significant when 1.96 times their standard error did not include zero (two-tailed test).

For tolerance analysis to be effective, each sire family should have both health and affected individuals. The sires had an average of 36% of their offspring affected (range, 9.4-89.7%, *n* = 83 sires), and 90% of the sires had 14.8% to 60.3% of their offspring affected. It is also good to note that sires and dams have brothers, sisters, and cousins in the data, and the animal model accounts for all these relationships in the genetic analysis, contributing further to a solid analysis.

### Covariance functions

Genetic and maternal (dam) variance of BW7 as a function of RATIOPlat trajectory was calculated: as *x*'*_RatioPlat_***G***x_RatioPlat_*, where $G=\left[\begin{array}{l} \sigma_{b0}^{2}\sigma_{b0b1}\\ \sigma_{b0b1}\sigma_{b1}^{2} \end{array}\right]$, and $\sigma_{b0}^{2}$ and $\sigma_{b1}^{2}$ are either genetic or maternal variances for intercept and slope, and $\sigma_{b0b1}$ is the respective covariance between these two terms (Kolmodin and Bijma 2004). The term *x_RatioPlat_* is a vector [1 *RatioPlat*]′ in which *RatioPlat* refers to a RATIOPlat value on the *x*-axis. The five separate environmental variances (*V*_E_) for BW7 combined with genetic (*V*_G_) and maternal variances (*V*_M_) estimated along RATIOPlat trajectory allowed the calculation of BW7 phenotypic variance (*V*_P_ = *V*_E_ + *V*_G_ + *V*_M_), heritability (*h*^2^ = *V*_G_ / *V*_P_), and maternal effect ratio (*m*^2^ = *V*_M_ / *V*_P_) as a function of heart ratio trajectory. Finally, following Calus *et al.* (2004), a genetic correlation between healthy birds (RATIOPlat = 0) and affected birds (RATIOPlat > 0) at a certain RATIOPlat value was calculated as: $r_{G}=\frac{x_{0}’Gx_{RatioPlat}}{\sqrt{x_{0}’Gx_{0}^{*}x_{RatioPlat}’Gx_{RatioPlat}}}$, where **G** is the genetic (co)variance matrix of slope and intercept, *x*_0_ is a vector of [1 0]’ for healthy animals with RATIOPlat of zero, and *x_RatioPlat_* is as described earlier. To ease the interpretation of figures, RATIOPlat values were back-transformed to the original heart ratio values.

## Results

### Genetic and maternal variation

The results revealed significant genetic variation for growth and heart ratio, an indicator of ascites resistance (Table 1). Body weights at 2 and 7 weeks had moderate heritabilities of 0.33 and 0.18, respectively. Heart ratio heritability was 0.34. These heritabilities had low standard errors, and all *h*^2^ estimates were greater than their standard errors. Maternal effect ratios for BW2, BW7, and RATIO were small but existent with estimates below or equal to 0.06 (Table 1). These results are similar to the previous estimates (De Greef *et al.* 2001; Moghadam *et al.* 2001; Pakdel *et al.* 2005), confirming that the data behaved in a solid expected way.

**Figure 1:**
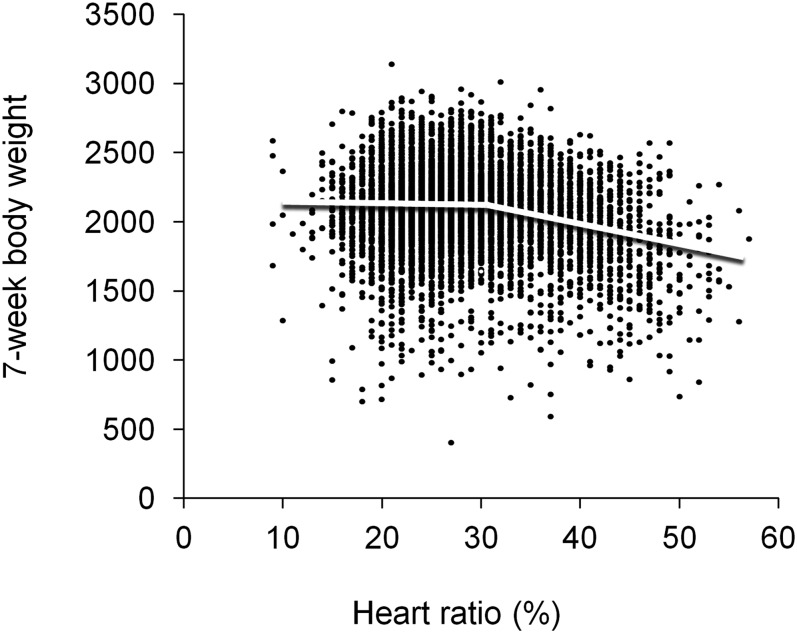
Phenotypic relationship between 7-week body weight and heart ratio. The plateau-linear regression for the population obtained from the statistical model 3 is drawn thought the data.

**Figure 2:**
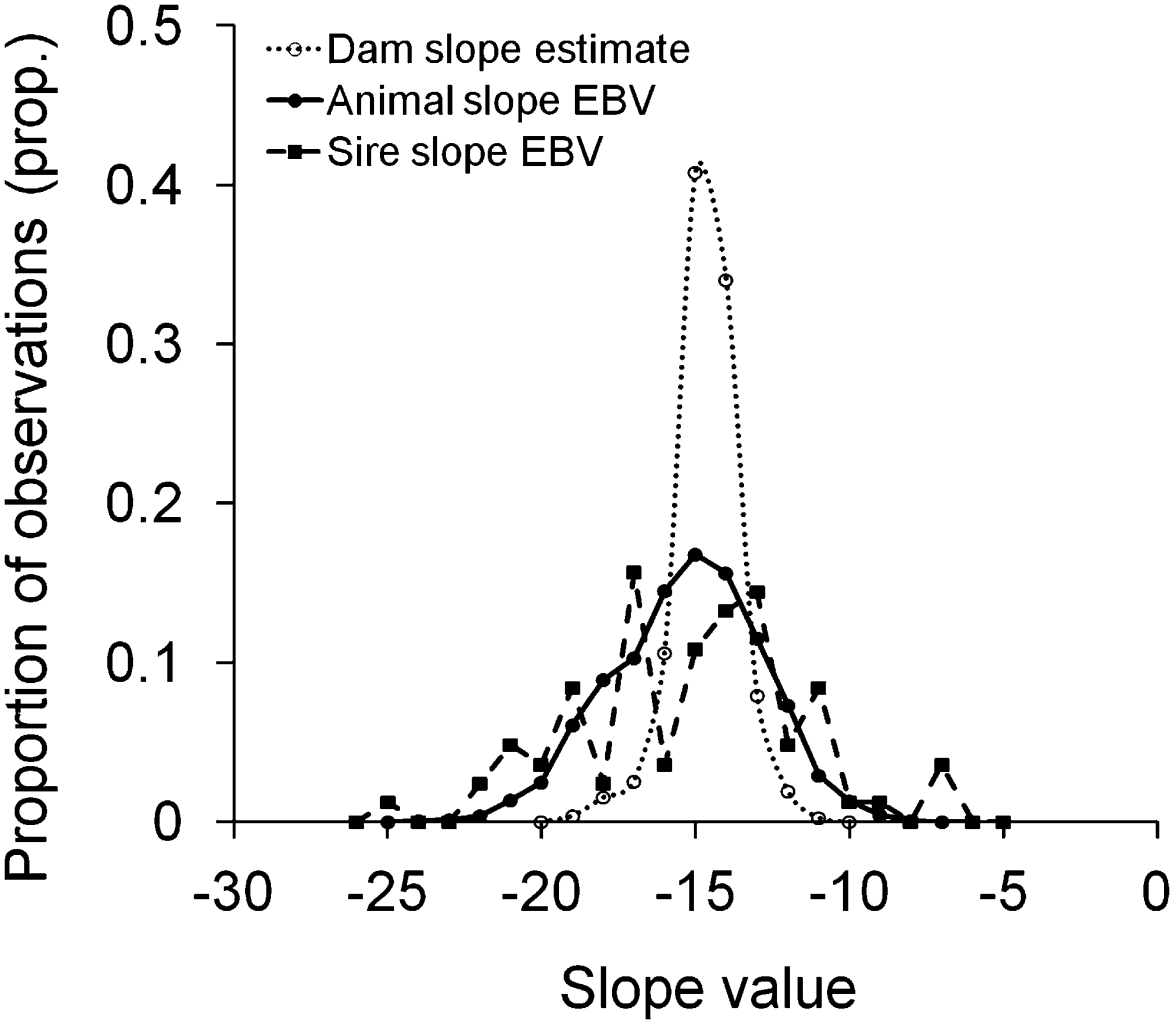
Frequency distributions of tolerance slope solutions for the maternal effect (dam slope estimate, *n* = 788 dams of the offspring generation), for EBVs of the animals in the offspring generation (animal slope EBV, *n* = 7722 offspring), and for EBVs of sires of the offspring generation (sire slope EBV, *n* = 83 sires).

The results revealed significant genetic variance for ascites tolerance. Genetic variance for tolerance slope was 57.8 and 59.3 for models either excluding or including BW2 as a fixed covariate term, respectively (Table 1). The variance estimates were 1.5 and 1.8 times greater than their standard errors, implying they were statistically significant. That the two models produced similar estimates implies a solid data structure for tolerance analysis, even without statistical correction for the initial growth performance (Kause 2011). Figure 1 introduces the average tolerance slope of the population, showing decreasing body weight with increasing heart ratio in affected birds. EBVs for tolerance slope for animals in the offspring generation ranged from −24.2 to −7.07 and for sires of the offspring generation from −24.9 to −6.97 (Figure 2). Some genotypes were hence less sensitive (weaker negative slope) while others were very sensitive (strong negative slope). Maternal effect tolerance slope solutions for the dams of the offspring generation ranged from −18.9 to −10.8. This reveals smaller variation for the maternal effect compared to the genetic effects (Figure 2) as expected based on the variance components (Table 1).

The standard error of maternal slope variance was of equal size to the variance estimate (Table 1). However, the dam slope effect was kept in the model because it is needed for covariance functions to calculate the significant maternal effect in BW7 (Table 1) along the heart ratio trajectory.

For the model excluding the BW2 covariate, genetic variance for INTERCEPT was 10640, that is, comparable with genetic variance of 12952 for BW7. Similar pattern was observed for maternal variances of INTERCEPT and BW7 (Table 1).

### Genetic correlations between body weights

Body weight at week 2 and 7 displayed moderate positive correlations (Table 2). Similarly, maternal and genetic correlations between BW2 and INTERCEPT were moderately positive (Table 3) and comparable with the correlations of BW2 and BW7 (Table 2). These are typical results for successive body weight measurements.

**Table 3:** Genetic (above diagonal) and maternal (below diagonal) correlations and their standard errors (± SE)

	BW2	RATIO	INTERCEPT	SLOPE
BW2		0.09 ± 0.11	0.71 ± 0.07	−0.27 ± 0.24
RATIO	−0.31 ± 0.22		0.15 ± 0.14	−0.36 ± 0.27
INTERCEPT	0.76 ± 0.09	0.003 ± 0.26		−0.30 ± 0.27
SLOPE	−0.005 ± 0.31	−0.80 ± 0.49	−0.26 ± 0.36	

### Trade-off between tolerance and resistance

Resistance and tolerance were genetically independent, implying a lack of genetic trade-off (Table 3). This was indicated by the nonsignificant weak genetic correlation between RATIO and tolerance slope (Table 3). The maternal correlation was strongly negative but with a very high standard error.

### Trade-off between tolerance and growth

Genetic and maternal correlations between tolerance slope and INTERCEPT were weakly negative but nonsignificant, implying that body weight of healthy birds was not related to the level of tolerance (Table 3). Likewise, maternal and genetic correlations of tolerance slope with BW2 were low.

### Trade-off between resistance and growth

No genetic trade-off was observed between resistance and body weight at 2 weeks of age (Table 2). The correlations of RATIO with BW2 were low, and 1.96 times their SE always included zero. Similarly, maternal and genetic correlations between RATIO and INTERCEPT (*i.e.*, BW7 in ascites-free birds) were weak and nonsignificant (Table 3). These results together indicate that ascites incidence was phenotypically and genetically independent of growth in ascites-free birds.

At 7 weeks of age, the correlations of RATIO with BW7, which includes the weights of both healthy and ascitic birds, were all moderately negative (from −0.23 to −0.33; Table 2). This shows that when the body weight of both healthy and affected birds are analyzed together, overall vigor is observed and birds genetically more prone to ascites have lower body weights. Phenotypic and genetic correlations of RATIOPlat with BW7 (*r*_P_ = −0.30; *r*_G_ = −0.42) were higher than the respective correlations of RATIO (*r*_P_ = −0.22; *r*_G_ = −0.33). This is expected when there is a nonlinear relationship between heart ratio and BW7 (Figure 1).

### Ascites-induced genotype-by-environment interactions

**Figure 3:**
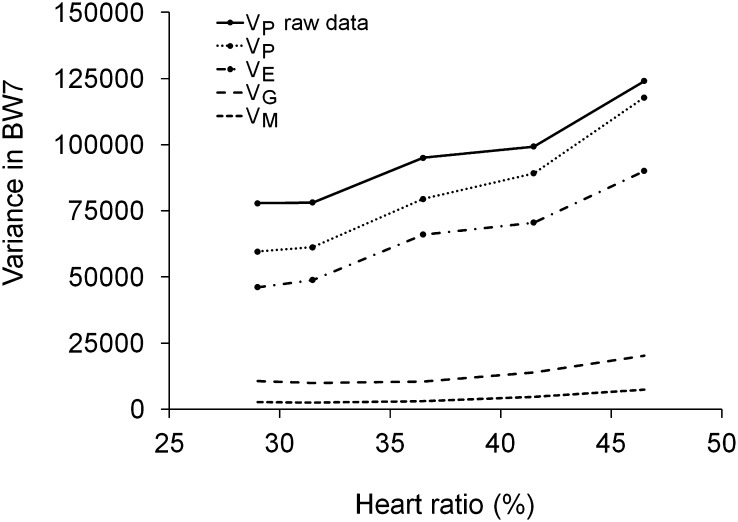
Phenotypic (*V*_P_), environmental (*V*_E_), genetic (*V*_G_), and maternal variance (*V*_M_) for 7-week body weight estimated using random regressions and covariance functions and phenotypic variance calculated directly from the raw data (*V*_P_ raw data) as a function of heart ratio.

Variance component estimates for 7-week body weight changed with increasing heart ratio (Figure 3). Phenotypic variance calculated from the raw data separately for the five *x*-axis classes along the RATIOPlat trajectory was elevated with increasing heart ratio. The phenotypic variance from the random regression tolerance model was lower compared with the phenotypic variance from the raw data because the fixed effects eliminated part of the variance. However, there was a tendency that the phenotypic variances were more similar at the high heart ratio end. This occurred because the maternal and genetic variances were elevated at the right-hand side of the trajectory, whereas the environmental variance was parallel with the phenotypic variance of the raw data (Figure 3).

Coefficients of phenotypic variation from the random regression model were 11.5%, 11.7%, 13.8%, 15.2%, and 19.1% at the five *x*-axis classes, showing that the increase in variance was not a consequence of a change in the BW7 mean.

**Figure 4:**
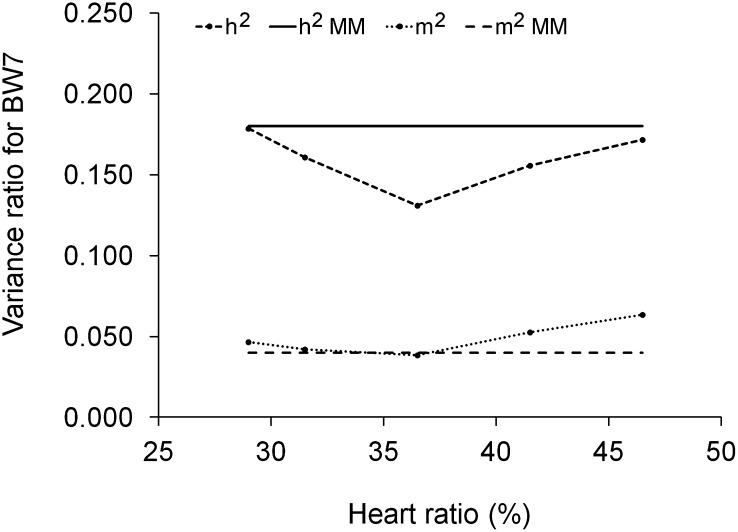
Variance ratios for 7-week body weight as a function of heart ratio. Heritability (*h*^2^) and maternal effect ratio (*m*^2^) were estimated using random regressions and covariance functions. Heritability (*h*^2^MM) and maternal effect ratio (*m*^2^MM) were estimated using mean model 1 (from Table 1).

In contrast to the variance components, there were only minor changes in heritabilities and maternal effect ratios of BW7 along the heart ratio trajectory (Figure 4). Moreover, the *h*^2^ and *m*^2^ estimates from the random regression model displayed a U-shaped trend along the heart ratio trajectory (Figure 4). The random regression estimates of *h*^2^ were equal or slightly lower, and the *m*^2^ estimates equal or slightly greater than the respective mean model estimates.

**Figure 5:**
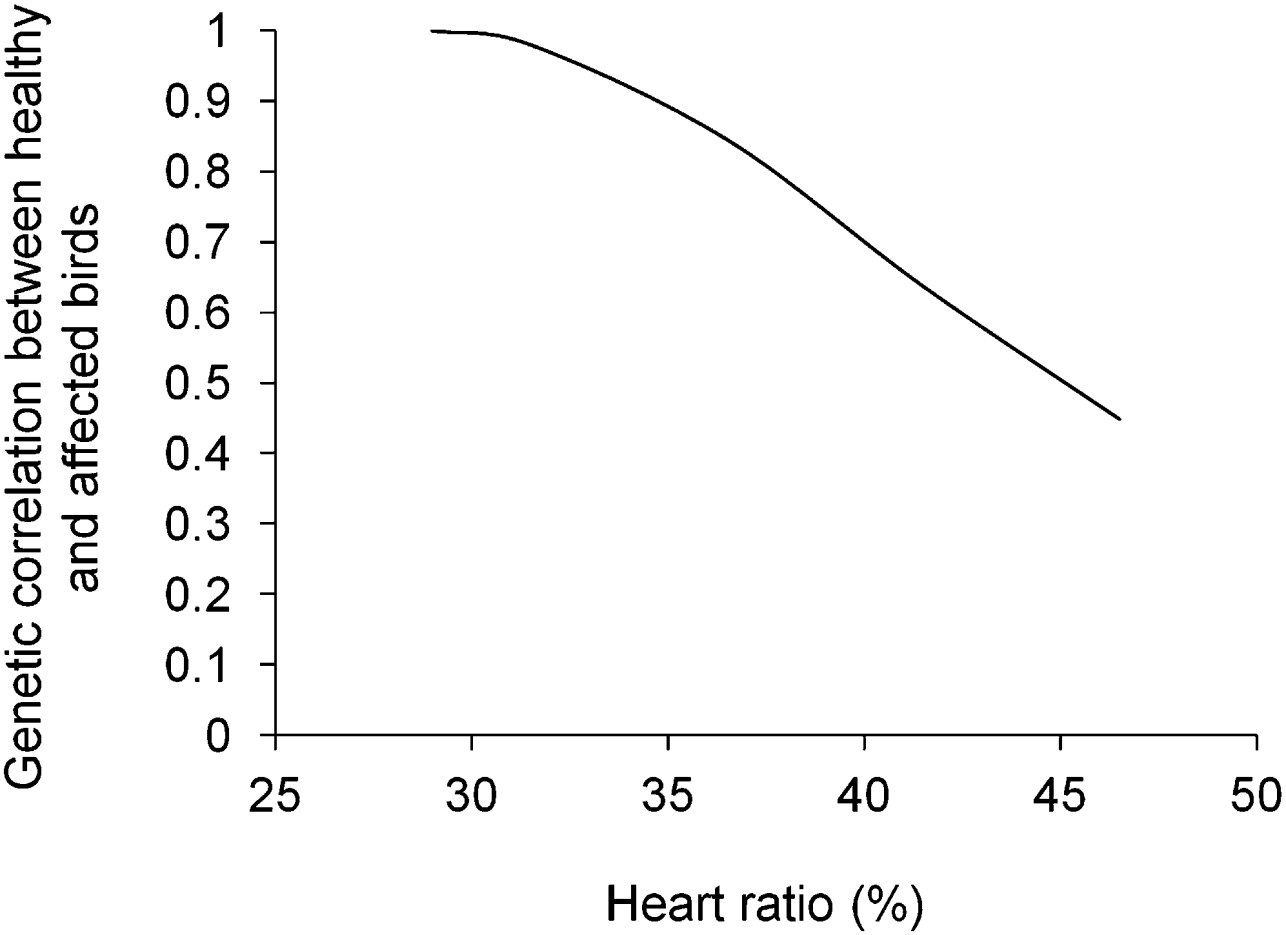
Genetic correlation between healthy and affected birds with different heart ratios (on *x*-axis), calculated using random regressions and covariance functions.

Genetic correlation between healthy and affected birds was reduced from unity to 0.45 at heart ratio of 46.5% (Figure 5), indicating extensive genotype re-ranking between healthy and severely affected birds.

## Discussion

### Random regression analysis of tolerance

The random regression methodology suggested by Kause (2011) was applied here to tolerance analysis. The present study demonstrated the merit of random regressions and covariance functions to estimate genetic variance for tolerance slope, its genetic correlations with other traits, and infection-induced genotype re-ranking and changes in genetic variation. By using an animal model, we were able to estimate EBVs for individuals even though tolerance itself cannot be recorded from an individual. The EBVs can be used for selecting genetically superior individuals but also in gene mapping studies. We applied the plateau-linear model of Ravagnolo and Misztal (2000a,b), yet an alternative would have been a mixture model analysis (Zerehdaran *et al.* 2006), which would assume heart ratio has two underlying distributions, one for healthy and one for affected birds whose growth is different affected. So far, the challenge in animal science has been that resistance and tolerance are difficult to uncouple, and the traits may be confounded in trait recording. For instance, whether an animal survives through a challenge test is in fact determined together by animal’s resistance and tolerance. Recently Ødegård *et al.* (2011a,b) developed a cure model to separate “susceptibility” and “endurance” from a challenge test data with time-until-death observations. These two concepts are comparable with the definitions of resistance and tolerance. Along the same lines, Árnason (1999) and Urioste *et al.* (2007) have proposed a bivariate linear-threshold model that can be used to analyze whether an animal survived (a threshold trait) and how long it took until death (a linear trait). These new statistical developments provide novel tools to increase our understanding of genetics of alternative strategies to fight against parasites, pathogen, and production diseases.

### Genetic variation for tolerance and resistance

The first major finding of our study was that both tolerance and resistance exhibited genetic variation. Tolerance to ascites displayed significant genetic variance, with the tolerance slope EBVs ranging from weakly negative (less sensitive genotype) to strongly negative (more sensitive), but no completely tolerant genotypes were observed. Resistance, measured as the heart ratio, had moderate heritability of 0.34. These results show that both resistance and tolerance are expected to respond to selection. Improved resistance and tolerance can be both used to reduce the harmful effects of ascites on birds.

In sheep, nematode tolerance is typically analyzed as the difference between body weight before and after a gastrointestinal nematode attack. This approach does not only analyze tolerance because it confounds both natural temporal variation in growth (*e.g.*, growth curves) and the impact of parasites on growth (Bisset and Morris 1996). Nevertheless, these studies are of great interest because of the lack of previous studies on genetics of tolerance to infections or production disease in animals. In sheep, resistance measured as fecal egg count of intestinal nematodes has higher heritability (*h*^2^ = 0.26-0.34) than two tolerance indicators, live weight gain depression (*h*^2^ = 0.09) and wool growth depression (*h*^2^ = 0.08), during an experimental challenge with nematode larvae (Albers *et al.* 1987). Heritabilities for tolerance measured as age at first drench and drench score were 0.13 and 0.14 in the study by Bisset *et al.* (1994) and 0.06 and 0.03 in the study by Bisset *et al.* (1996), reflecting again that tolerance may have only modest genetic variation in sheep. Drenching reflects tolerance because it was applied only to the animals whose live weight change was below an acceptable threshold. In dairy cows, individual variation exists for loss in milk yield in response to experimentally induced *Escherichia coli* mastitis (Vandeputte-Van Messom *et al.* 1993). Different inbred mice strains display differences in tolerance against malaria *Plasmodium chabaudi* (Råberg *et al.* 2007).

Compared with animals, studies on genetic variation in tolerance to infections are plentiful in plants (Fineblum and Rausher 1995; Tiffin and Rausher 1999; Koskela *et al.* 2002; Kover and Schaal 2002; Carr *et al.* 2006; and references therein). For instance, *Arabidopsis thaliana* accessions show genetic divergence in tolerance to bacteria *Pseudomonas syringae* (Kover and Schaal 2002). Common monkey-flower *Mimulus guttatus* displays close-to-zero heritability (*h*^2^ < 0.03) for both resistance and tolerance to *Cucumber mosaic virus*, tolerance measured as the difference between a pair of control and infected individuals (Carr *et al.* 2006). Stinging nettle *Urtica dioica* displays family differences in tolerance to holoparasitic Dodder *Cuscuta europaea* (Koskela *et al.* 2002).

In our study, tolerance heritability could not be estimated because the data included only one record per individual. Heritabilities for tolerance regression parameters can be estimated when each individual has several performance observations. By using regression slopes of individuals as raw observations in the genetic analysis, both environmental and genetic components of slope variance, and hence also heritability, can be estimated (Schaeffer 2004).

### Trade-off between tolerance and resistance

The second major finding was that resistance was genetically independent of tolerance, and thus the traits did not display a genetic trade-off. Simultaneous genetic improvement of ascites resistance and tolerance is hence possible.

In line with our study, the meta-analysis of 31 studies on 17 plant species demonstrates that in general tolerance and resistance to herbivores are weakly genetically correlated (Leimu and Koricheva 2006). Albers *et al.* (1987) found in sheep favorable positive genetic correlations (*r* = 0.31-1.00) between resistance (fecal nematode egg count) and tolerance (weight gain and wool growth depressions) against nematodes, but three of four correlations were nonsignificant with large SEs. Piper and Barger (1988) showed in sheep that offspring of sires suffering the greatest nematode burdens also tended to suffer the greatest production losses under a nematode attack. In contrast, Bisset *et al.* (1994; 1996) found no genetic relation between resistance (fecal egg count) and tolerance (drenching score) to nematode parasites in Romney sheep (*r* = −0.18 to 0.21). These results together imply no genetic trade-off between resistance and tolerance.

The analysis of five inbred mice strains showed a strong genetic trade-off between resistance and tolerance to malaria (Råberg *et al.* 2007). Correlations across distinct inbred strains do not need to be consistent with within-population genetic correlations.

### Trade-off between tolerance and growth

The third major finding here was the nonsignificant genetic correlations of tolerance slope with body weights in ascites-free birds. In other words, healthy birds with high body weight were not genetically more prone to a strong reduction in body weight as the result of ascites compared with the healthy birds with a lower body weight. Both the theory (Kolmodin *et al.* 2003; van der Waaij 2004) and some observations (*e.g.*, Ravagnolo and Misztal 2000a) imply that well-performing individuals are genetically more sensitive to changes in an environment. No evidence for such a genetic trade-off was found here for tolerance and body weight. These results imply that simultaneous breeding for both increased growth and ascites tolerance is possible.

In the review by Núñez-Farfán *et al.* (2007), 8 of the total 9 studies on plants showed a genetic cost of tolerance, typically in terms of reduced seed or fruit production. Nonetheless, the costs have been observed to be environment dependent (Núñez-Farfán *et al.* 2007). In broilers, studies with additional traits or in more deprived environmental conditions might reveal genetic trade-offs for ascites tolerance.

### Trade-off between resistance and growth

The fourth major finding was that the correlation structure between ascites resistance and body weight was labile. Even the sign of the correlation was switched implying that the presence or absence of a disease creates a labile correlation structure. The expression of phenotypic or genetic costs of resistance varied depending on the age of the birds and ascites incidence in a population. A cost of resistance is fundamental for the theories of maintenance of genetic polymorphism (Boots and Bowers 1999; Best *et al.* 2008), but the expression of costs may be more labile than assumed by the models.

The likely explanation for the labile correlation is that the incidence of infected individuals and the severity of performance reduction due to ascites can influence the sign of the correlation. The simulation by Zerehdaran *et al.* (2006) showed that growth under conditions of no ascites (or early growth) can be weakly or not correlated with ascites incidence. This is because growth retardation has not yet influenced growth performance of individuals. In contrast, the sign of the correlation is switched to negative when ascites incidence is increased (or in older animals with more severe symptoms) because the affected individuals suffer from reduced growth (Zerehdaran *et al.* 2006).

The model by Zerehdaran *et al.* (2006) is supported with the real data. Genetic correlation between ascites and 35-d body weigh in nonaffected broilers is positive (*r* = 0.29), whereas the correlation in the whole population with both affected and nonaffected individuals included is negative (*r* = −0.26) (de Greef *et al.* 2001). Such a change is in line with our observations that phenotypic and genetic correlations of ascites resistance with initial 2-week body weight were 0.04 and 0.12 but with 7-week body weight −0.23 and −0.33, respectively. Likewise, the genetic correlation of heart ratio with body weight of ascites-free birds (trait INTERCEPT) was 0.15. A similar change in correlation structure during growth occurs for skeletal deformations (Kause *et al.* 2005) and cataract induced by a parasite in rainbow trout (Kuukka-Anttila *et al.* 2010).

Consequently, testing for a relationship between host performance and ascites, or any disease reducing host performance, is challenging because the incidence of infected animals and the severity of host performance reduction can influence the sign of the correlation. This creates variability across studies in the correlations. Thus, the question is whether there are certain regularities in the manner in which the genetic trade-offs vary and evolve (Kause *et al.* 2001; Kause and Morin 2001) and which factors create such variation (Núñez-Farfán *et al.* 2007). The present study stresses the fact that diseases can induce changes in correlations, leading to environment-dependent correlated genetic responses to selection.

### Disease-induced genotype-by-environment interactions

The final major finding was that ascites induced genotype-by-environment interactions in body weight. Phenotypic and genetic variance of body weight was increased with increasing heart ratio. Similarly, Pakdel *et al.* (2005) and Zerehdaran *et al.* (2006) observed higher phenotypic coefficients of variation in body weight for broilers held in cold compared to broilers held in warm temperature. In the present study, the change in body weight heritability was very modest, the heritability estimate ranging from 0.13 to 0.18. Pakdel *et al.* (2005) found heritabilities of 0.50 and 0.42 with overlapping confidence limits in 5-week body weight for broilers held under warm and cold conditions, respectively.

Two nonmutually exclusive explanations exist for the observed change in body weight variation. First, diverging tolerance slopes increase genetic and nongenetic variance of body weight with increasing ascites severity. Body weight variation is elevated because growth of birds is differently influenced by ascites. Second, random regressions can artificially create an increasing or u-shaped variance pattern across an *x*-axis (Ravagnolo and Mistzal 2002b; Kause 2011). Because even the raw phenotypic variance displayed an increasing trend, the latter explanation does not fully explain the observed trend here.

Infections can change heritabilities of performance traits. For instance, Lewis *et al.* (2009) showed in pigs elevated heritabilities for reproduction traits in response to porcine reproductive and respiratory syndrome outbreaks. Charmantier *et al.* (2004) showed reduced heritability of tarsus length in Blue tit (*Parus caeruleus*) under blowfly larvae attack. Kause *et al.* (2007) showed increased liability-scale heritability for skeletal defects as a function of increasing defect incidence.

Ascites did not just induce a scaling effect (a change in variance), but also genotype re-ranking across healthy and affected birds. The most extreme genetic correlation between healthy and affected birds was 0.45. Pakdel *et al.* (2005) found even stronger genotype-by-environment interaction for body weight of broilers held under cold and warm temperature (*r*_G_ between environments = 0.28). The two forms of genotype-by-environment interaction, scaling effect and re-ranking, facilitate environment-dependent genetic changes. Moreover, when genetic correlations between host performance and resistance or tolerance are changed in response to infection, as shown here, the genetic outcome of selection in environments with differential pathogen burden becomes even more multifaceted.

Ascites is a clear example in which selection in disease-free and diseased population would result in different genetic responses in host performance and ascites resistance/tolerance. The modification of genetic architecture of growth and life-history traits by pathogens, parasites, and production diseases, mediated by tolerance genetics, may play a more fundamental role in microevolution than was previously though.

## Supplementary Material

Supporting Information
