# Supplementary material for: Genetics of Ascites Resistance and Tolerance in Chicken: A Random Regression Approach
Source: G3 (Bethesda). 2012 May 1;2(5):527–35. doi: 10.1534/g3.112.002311 (PMC3362936; doi:10.1534/g3.112.002311)
Supplement: Supporting Information [file supp_2_5_527__index.html]

Supporting Information 

# Genetics of Ascites Resistance and Tolerance in Chicken: A Random Regression Approach

## Supporting Information for Kause, van Dalen, and Bovenhuis, 2012

**Files in this Data Supplement:**

- File S1 - Supporting Data (.zip, 117 KB)
